# New York Hospital

**Published:** 1840-01-01

**Authors:** 


### NEW YORK HOSPITAL.

### REPORT OF CASES OF UNUNITED FRACTURE TREATED AT THE HOSPITAL. By JOHN S. HEARD, M.D. late House-Surgeon.

Passing over an enumeration of the various modes of treating ununited fracture that have been resorted to, Dr. Heard describes the following, and pins to it some remarks which we shall quote.

*Removing the extremities and connecting the fragments by silver wire.*—All these different methods having occasionally failed in effecting bony union,—*it occurred to Dr. J. Kearny Rodgers, one of the surgeons of the New York Hospital, that if, after removing the extremities of the fragments and exciting their periosteum, he could keep them in apposition for a certain period,—firm osseous union would ensue.* He accordingly after sawing off the extremities of the fragments, drilled a hole in the end of each, and passing a silver wire through, drew the bones together, and kept them in co-aptation until firm union was effected. Subsequently, Drs. Mott and Cheesman, colleagues of Dr. Rodgers, were likewise successful in inducing bony union by the same operation. This operation has not, we believe, been adopted in any other place than the New York Hospital. Indeed, the only notice of it that I have been able to meet with, is in a report of a clinical lecture by Mr. Liston, in which he makes these remarks : 'A proposal has, I think, been made, to denude the ends of the bones, drill holes in them, and connect them by a silver wire. It is too artificial a mode of proceeding, and I should fear, that from exposure and bruising of the ends necessary for accomplishing the object, (for perforation cannot be made unless by denuding the bone freely, and holding it securely by some contrivance until the object is accomplished,) that necrosis, instead of union, would be apt to follow.' Now, what Mr. Liston means by 'too artificial a mode of proceeding,' is not very apparent : in what is it more artificial than Mr. White's method? In the simple passing of a silver wire: and is an operation, although successful, to be abandoned because it is artificial? Surely this rule would have a very limited application in surgery. And as regards 'necrosis instead of union,' being more likely to follow, it will be sufficient to state, that this operation has hitherto never failed of success. And are we to refuse to adopt a sure plan of treatment, although it be severe, for one which, though less so, still, occasionally, is of no avail?"

Dr. Heard reports five successful cases and one unsuccessful, in which this operation was performed. Whatever the à priori objections to it, the facts deserve consideration.

---

### MR. MALCOLMSON'S CLINICAL OBSERVATIONS ON SLOUGHING OF THE ABDOMINAL PARIETES, CONNECTED WITH ABSCESS OF THE LIVER.*

Mr. Malcolmson observes :—

"Mr. Cæsar Hawkins, surgeon to St. George's Hospital, London, has published an elaborate paper of eighty pages, in the eighteenth volume of the Transactions of the Royal Medico-Chirurgical Society, on what he styles 'Sloughing Abscess connected with the Liver,' in which the gangrenous ulceration of the integuments and other parts around the opening made into the abscess in two of his patients, is ascribed to the peculiar or malignant nature of the disease ; and from this and other circumstances, he endeavours to establish the conclusion, that these cases were not examples of abscess of the liver, but of encysted

---

* Edinb. Med. and Surg. Journ. Oct. 1839.

tumors of the peritoneal coat. I have, however, found this gangrenous ulceration of the parietes of the abdomen to occur in several cases of common abscess in the substance of the liver, and believe it to depend on a cause quite unconnected with any thing peculiar in the internal disease, and that it becomes a serious obstacle to the recovery of many patients in whom ordinary abscesses of the liver are opened. These views are mentioned in a paper, which the same Society did me the honour to publish in the last volume of their Transactions. But although the symptom in question occurred in the remarkable case there narrated, it is not to be expected, that an opinion advocated by so experienced a surgeon as Mr. Hawkins, and adopted in other publications, should be overthrown by an incidental remark in a paper devoted to other objects. I shall, therefore, take this opportunity to relate several cases in which gangrenous ulceration and sloughing of the edges of the wound occurred, and in which dissection showed the disease to be ordinary abscess in the liver."

Mr. Malcolmson relates several cases, some more, some less to the point. Certainly, however, he proves his case, and satisfactorily establishes the occurrence of phagedænic ulceration or gangrene of the abdominal integuments as consequence of common abscess in the liver.

We shall quote one case, not because it is the most conclusive, but because is the shortest.

*Case.*—A field officer, many years in India, and who had lived very freely, was admitted with a great swelling, like a child's head, in the epigastrium, extending nearly to the umbilicus; it was hard, except in the centre, where there was fluctuation and a violent arterial pulsation. The patient was early sensible of a fluttering in the side, and he was the first to detect the existence of fluid, the tumour having been at first hard, fixed, and insensible; he had been for some years subject to hepatic disease, and (according to report) to irregular action of the heart and arteries. He was on the sick-report only three weeks previous to his removal to this station in consequence of the march of his regiment; and early in his illness, the bleeding from four leeches produced collapse, from which he did not recover for eight hours. By my advice, an opening was made, from which fourteen ounces of reddish purulent matter, with thick diseased masses and sloughy membranes, were evacuated, the greater part of the latter remaining attached internally. The mind became confused, the wild ideas being of a pleasing kind and not constant. There was a purulent-like deposit in the urine, which, on drying, afforded crystals of a light pink-colour. He sank in a few days, sloughing of the parietes of the abdomen, round the wound, commencing in a leech-bite, having previously made some progress.

On inspecting the body, the abscess was found in the convex surface of the liver, which was attached to the diaphragm and anterior part of the peritoneum, and had pushed the stomach downwards and to the left side. The abscess in the substance of the liver was of moderate size, and contained good purulent matter, but a secondary collection of matter had formed within the adhesions, in which the peritoneum was found in a state of slough, protruding in shreds through the wound, the edges of which were gangrenous. The left lobe was hard, and contained tubercles; the gall-bladder contained good bile.

Enough of this. We introduce another observation or two of Mr. Malcolmson's. And first of the *disregard of medical opinions evinced by commanding officers in the army.*

"This patient like the last fell a victim to marching during May and June, the hottest months of the year, in a climate the mean annual temperature of which is 80°; and these months are 10° and 13° above this respectively; with 18° to 21° mean diurnal range, the maximum being a little above 100°, while the minimum seldom falls below 90°.* At this time, no troops were allowed to march till half-an-hour after sunrise, in consequence of an opinion adopted in certain quarters, that cholera, which is so frequently destructive to troops on the march, is caused by miasmata which would be dispersed by the heat of the sun; an opinion not entirely without foundation, but which should never have been adopted without reference to season, particular climate, and other circumstances, and which proved most disastrous to this small detachment of young soldiers, several others of whom had their health permanently destroyed by liver disease contracted during this march. It is so seldom that military authorities will attend to the best considered medical advice, that too much caution cannot be observed by those who have an opportunity of offering it; nor would it have been difficult to have foreseen the effects of marching European troops on foot, in woollen clothes, and loaded with arms from half-an-hour after sunrise, till 9, 10, or 11, A. M.; and who had then to wait till their tents came up, an hour or two for breakfast, of which they partook when exhausted by fatigue, thirst, and the direct rays of a burning sun and soil. Nor are the ill effects of marching at such a season entirely done away with, by avoiding this extreme degree of exposure, as I have had twice an opportunity of witnessing when marching in charge of European troops during the hot winds. It is melancholy to think that European soldiers should, in times of profound peace, be exposed at such seasons, which the natives themselves avoid; but it is some consolation to know, that the regular exercise, and greater temperance usual on a march, with the dryness of the air, and of the ground on which the men sleep, do not tend to the production of disease; and that marching in the rainy season, especially in the basaltic districts, where the soil is deep and retentive of moisture, and fever is usually prevalent, is even more injurious. Of this a remarkable example occurred to me in 1828, in six men who rejoined the horse and foot artillery at Nogpore from the coast, and who were all admitted into the hospital on their arrival; and their future history was brought to the notice of the military authorities, in the end of 1829, as follows; 'Four never recovered, one died in May 1829; one has been discharged the service; another has been sent to the coast on sick leave; and the fourth will probably be discharged next committee. The remaining two have had very frequent relapses since; and the non-commissioned officer sent with the party arrived labouring under severe fever.' An order was in consequence issued, prohibiting men from being sent through the jungles during the rainy months; in which it is to be regretted that the season of the hot winds was not included. So much more depends on the prevention of disease in hot climates, than on the most judicious treatment, and it is so seldom that facts such as may make an impression on non-professional men occur, that this digression will not be thought out of place, and was indeed one reason for selecting these two cases for illustration."

Our readers need not be told that the profession has been too much snubbed at head-quarters. Witness the Army Medical Reports—witness the Navy—witness (on land) the Poor-law Commissioners, and their remuneration of medical men. Aide toi et le ciel t'aidera must be the motto of the profession. They will only receive consideration and respect when they assume a disagreeable attitude.

Mr. Malcolmson makes some observations on the use of mercury in hepatitis, which we cite without comment.

" These and innumerable other cases clearly show how erroneous the notion is, still very generally prevalent, that an extreme torpor of the absorbents exists in severe hepatitis, dysentery, and remittent fever, and that this is the cause 'why the largest doses internally, and the most assiduous inunctions externally,

* "Journal of the Asiatic Society of Bengal, Vol. ii. p. 239 and 542."

will sometimes fail in introducing a sufficient quantity of mercury to saturate the system.'* And hence the use of calomel in scruples and drachms daily, and rubbing in mercurial ointment, without reference to quantity, under the idea that all that has been taken or rubbed in before has not entered the system, and can therefore have no effect on the disease or the constitution. But if it be true that the mercury is absorbed, and mixes with the circulating fluids, how injurious must such practice be, when the medicine employed is one of such unequalled value in these diseases, but at the same time so energetic in its effects on the various tissues and organs, and whose mischievous effects remain so long in the constitution that has been thus poisoned with it; and how little to be wondered at is it, that many practitioners run into the other extreme of discarding it altogether, or of combining it with a severe and mischievous course of purgation, that may indeed sometimes obviate these evils, but, at the same time, take from our hands the power of using it effectually, and in regulated quantity; and proves directly injurious by irritating the intestinal canal, of itself (in such cases) too liable to take on disease, and by depressing the powers of the constitution in a greater degree than the freest and most effectual depletion. Considering this question as one of vital importance, and without satisfying myself regarding which I could not practice in India with any satisfaction, I was not content with the study of my own cases, but obtained and carefully analysed the records of some other hospitals, the intelligent physicians of which were more or less influenced by the notions referred to; and I found, that when great quantities of mercury had been unsuccessfully employed, (with the exception of extremely few cases, that might be explained by imperfection in the records, or by idiosyncrasy,) the gums became more or less sore after the exhibition of very moderate quantities of the medicine, and that in general a slight and evanescent ptyalism took place very early, showing clearly that it did enter the system, and that its usual sensible effects were prevented by the existence of disease overpowering its action. When, therefore, the gums or salivary glands are at all affected by moderate doses of mercurial medicines, and when during their continuance the partial salivation becomes less marked, and the gums less tender, or when the effect of the medicine does not become more evident, or when it too rapidly diminishes on its being omitted, it may be certainly concluded that it will not affect the system in a beneficial manner until an impression is made on the disease by other means, and that it is always necessary to stop its exhibition when a moderate quantity has been prescribed.

It is the irritation of the disease, as above stated, that prevents the mouth becoming affected in liver disease, in dysentery, in peritoneal inflammation, and still more in fever, in all of which there are abundant proofs of the mercury having been absorbed, in the severe salivations, loss of teeth, pains of the limbs, and affections of the periosteum, that too often follow its employment, especially in those cases in which unsuccessful attempts have been made to affect the mouth in the early period of the disease. It is, however, necessary to be aware, that a slight degree of irritation is sufficient to stop salivation for a time, even when fully established, and that, therefore, it may not always be proper to omit the medicine, until it has had a fair trial. Thus the paroxysm of an intermittent will sometimes dry up the mouth during the hot fit; and I have seen the same happen from the excitement of a blister. Hence the observant practitioner must not omit to use any assistance to be derived from the peculiar appearance of the gums, the character of the saliva, and other means by which his judgment may be assisted; it will, however, seldom happen, that, when depletion

---

* "I have had a couple of Malays daily employed for hours at a time in unsuccessful frictions, the lymphatic system refusing to take up the ointment in any considerable quantity.—*Johnson on the Influence of Tropical Climates.*"

has been as freely used as the case will permit, mercury can be safely continued, when its effects on the mouth either diminish or do not advance under the use of moderate doses. These remarks are also applicable to a rule insisted on by some authors, that a sore mouth is useful as an index of the system being impregnated with mercury, and should therefore, if possible, be induced."

